# Combined detection of miR-21-5p, miR-30a-3p, miR-30a-5p, miR-155-5p, miR-216a and miR-217 for screening of early heart failure diseases

**DOI:** 10.1042/BSR20191653

**Published:** 2020-03-18

**Authors:** Han Ding, Yin Wang, Longgang Hu, Sheng Xue, Yu Wang, Lei Zhang, Yuan Zhang, Hongzhao Qi, Hua Yu, Lynn Htet Htet Aung, Yi An, Peifeng Li

**Affiliations:** 1 Institute for Translational Medicine, Medical College, QingDao University, Qing Dao 266071, China; 2 The Affiliated Cardiovascular Hospital of Qingdao University, Qng Dao 266071, China

**Keywords:** Combined detection, diagnostic biomarker, early heart failure diseases, microRNAs

## Abstract

The use of circulating microRNAs as biomarkers opens up new opportunities for the diagnosis of cardiovascular diseases because of their specific expression profiles. The aim of the present study was to identify circulating microRNAs in human plasma as potential biomarkers of heart failure and related diseases. We used real-time quantitative PCR to screen microRNA in plasma samples from 62 normal controls and 62 heart failure samples. We found that circulating miR-21-5p, miR-30a-3p, miR-30a-5p, miR-155-5p, miR-216a and miR-217 expressed differently between healthy controls and heart failure patients. Plasma levels of miR-21-5p, miR-30a-3p, miR-30a-5p, miR-155-5p, miR-216a and miR-217 were unaffected by hemolysis. Correlation analysis showed any two of these miRNAs possess a strong correlation, indicating a possibility of combined analysis. MiR-21-5p, miR-30a-3p, miR-30a-5p, miR-155-5p, miR-216a and miR-217 could be combined in two or three or more combinations. The results suggest that miR-21-5p, miR-30a-3p, miR-30a-5p, miR-155-5p, miR-216a and miR-217 may be a new diagnostic biomarker for heart failure and related diseases.

## Introduction

In recent years, cardiovascular diseases still rank the first killer of human death and heart failure accounts one of them. Heart failure is not an independent disease but the end stage of the development of heart diseases. Although the trend of younger heart failure group rises substantial in recent years, no enough research attention has been paid to the younger generation [1–5]. Therefore, effective diagnosis and prevention of heart failure is an urgent problem in medical and biological researches [6–9].

MicroRNAs (miRNAs) are a class of non-protein encoded small RNAs that widely exist in eukaryotes and have a length of 21–25 nucleotides. They are highly stable in the blood circulation and can regulate gene expression in a sequence-specific manner. They play an important role in development, apoptosis, metabolism and human diseases. The physiological and pathological regulation mechanism of miRNA is a new discipline which has been highly valued in recent years [10–12].

Recent studies have shown that cardiovascular diseases can cause significant changes in the expression level of specific miRNAs in the body. Therefore, detection of specific miRNAs in body fluids can play an essential role in the diagnosis and prevention of cardiovascular diseases [13–16]. However, there is still insufficient research on specific miRNAs in heart failure [17–20].

Several studies showed that the dynamics of many miRNA expression are closely related to the occurrence of diseases; for example, many heart failure-related miRNA expression changes *in vivo* related to the occurrence of heart failure. To this end, we selected six miRNAs from previous studies, namely, miR-21-5p, miR-30a-3p, miR-30a-5p, miR-155-5p, miR-216a and miR-217, which showed an upward trend with the occurrence of heart failure. In combination with other researchers ‘statistics on sample size, we selected more than 60 healthy samples and more than 60 cases of heart failure. Exhaustion samples were analyzed [21–38].

**Figure 1:**
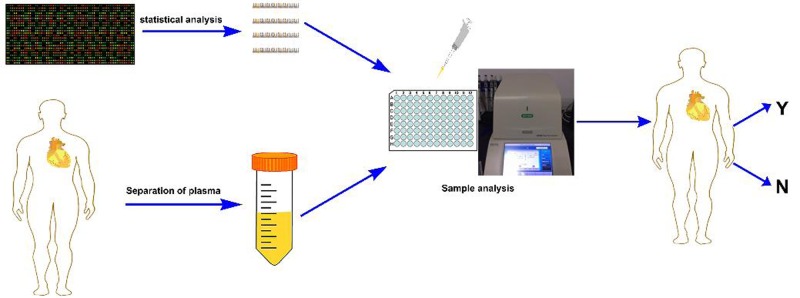
Schematic maps of screening and identifying microRNAs as potential biological targets for heart failure detection Among them, Y means that the test result is positive, and N means that the test result is negative.

As shown in Figure 1, the aim of the present study was to identify circulating miRNAs in human plasma as biomarkers for the diagnosis of heart failure and its related diseases, to assess the appropriate biomarkers for identifying key characteristics of miRNAs, and to analyze their performance.

## Materials and methods

### Study population

Patients with heart failure were recruited at the Affiliated Hospital of Qingdao University, Qingdao, China. The healthy control group was from the physical examination center of Qingdao University affiliated hospital. Through similar experiments by other researchers, we finally identified more than 60 healthy control samples and more than 60 heart failure disease samples. Detailed characteristics of heart failure group and control group were listed in Table 1.

**Table 1:** Partial indexes of heart failure group and healthy control group

Variable	Heart failure	Control	*P*-value
	*N* = 62	*N* = 62	
Age (years)	62 ± 8.89	60 ± 11.80	0.55
Men	40	42	
BMI (kg/m^2^)	26.07 ± 3.36	24.76 ± 3.83	0.13
Total cholesterol (mg/dl)	4.57 ± 1.56	4.08 ± 1.51	0.23
Triglycerides (g/l)	1.62 ± 0.81	1.14 ± 0.67	0.0047**
LDL cholesterol (mg/dl)	2.86 ± 1.41	2.52 ± 1.12	0.29
HDL cholesterol (mg/dl)	1.15 ± 0.29	1.05 ± 0.53	0.49
Creatinine	62.7 ± 16.76	76.0 ± 18.46	0.0018**
NT-proBNP	1809.6 ± 1465	100.1 ± 82.36	0.0384*
CKMB	12.7 ± 27.46	11.8 ± 8.93	0.87
MYO	245 ± 441.16	40.4 ± 13.35	0.36
HsTnT	0.467 ± 1.16	0.005 ± 0.0055	0.43

BMI: body mass index; CKMB: creatine kinase-MB; HDL: high-density lipoprotein; HsTnT: hight-sensitivity troponin T; LDL: low-density lipoprotein; MYO: myoglobin; NT-proBNP: N-terminal pro-B-type natriuretic peptide. Data are shown as mean±SE; *P<0.05 and **P<0.01.

### Blood collection

Peripheral blood from patients with heart failure and healthy people was collected in 10 ml EDTA anticoagulation tube. After 30 min of blood collection, the samples were centrifuged at 3000 ***g*** and 4°С for 10 min. After centrifugation, the plasma was separated and frozen immediately until RNA was separated.

### miRNA extraction

RNA isolation was advanced by using the traditional Triazole method. The specific method was: 250 μl plasma was mixed with 750 μl Triazole, then 10 μl and 50 pM of coccidian RNA was added. In the experiment, the RNA of nematode was used as the standard external reference, and 200 μl chloroform was added to the plasma and mixed with severe vibration for 15 s. Centrifuge for 10 min at the condition of 4°С and 12,000 ***g***, removed 550 μl from the lower layer and added isopropanol of the same volume, added 5 μl glycogen, mixed upside down, rest overnight at the condition of −20°С, centrifuge for 10 min at the condition of 4°С and 12,000 ***g***, discard the supernatant, added 75% ethanol without RNase enzyme of 1 ml into the tube, and separated at the condition of 4°С and 12,000 ***g***. After 10 min, the supernatant was discarded and 75% ethanol without RNase enzyme was added into the tube. The supernatant was discarded and then centrifuged for 5 min at 4°С and 12,000 ***g*** for 10 min at room temperature. Water with 20 μl of Ranse without enzyme was added into the tube and dissolved at 4°С. The RNA was quantified and quantified by RNA electrophoresis gel and measuring concentration.

The extracted RNA was inverted by TaKaRa, the Mir-X miRNA First-Strand Synthesis Kit (Clontech). The specific method of inversion was the standard method of TaKaRa reverse transcription.

### Quantitative PCR experiment

We selected TaKaRa quantitative PCR kit and Bio-Rad real-time quantitative PCR instrument to process and analyze the samples we collected. According to the instructions of TaKaRa products, the instrument was programmed to react in three steps, including the first step of 95°С reaction for 15 min, the second step of reaction including 94°С reaction for 20 s, 63°С reaction for 30 s, 72°С reaction for 34 s, the whole process was repeated five times, the third step of reaction including 94°С reaction for 20 s, 63°С reaction for 34 s and collection. The fluorescence signal was repeated 45 times, and then the reaction was completed after 5 s of 65°С reaction and 5 s of 95°С reaction. All data were repeated three times in the experiment.

### Data analysis and statistical methods

Quantitative miRNA expression data ABI SDS software (Life Technologies) was used to estimate the cyclic threshold (CT) with an average threshold of 0.2 (30). The CT value > 35 is considered below the detection limit [39] for calculation, the CT value > 35 is labeled 35 [40]. The level of miRNA is 2^− Ct^ [41].

Then, we used SPSS software, MedCalc software, Prism software to further analyze the data of fluorescence quantitative PCR.

## Results

### Expression profile analyses of candidate miRNAs

**Figure 2:**
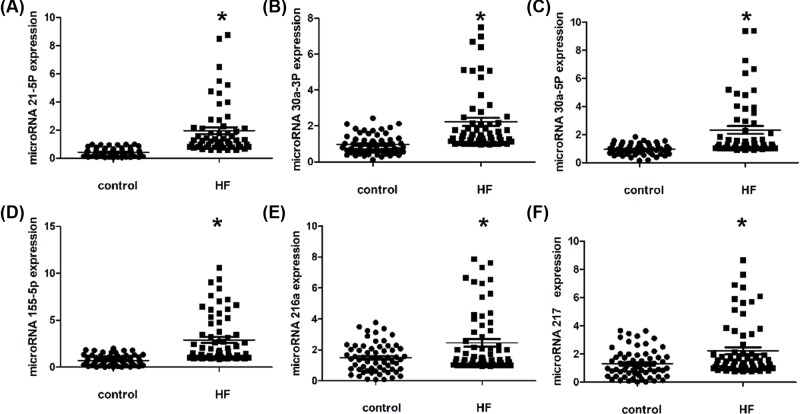
Plasma miRNA levels in the validation population The scatter plots show the expression levels of (**A**) miR-21-5p, (**B**) miR-30a-3p, (**C**) miR-30a-5p, (**D**) miR-155-5p, (**E**) miR-216a and (**F**) miR-217 measured by quantitative real-time polymerase chain reaction (qRT-PCR) in patients with HF and control subjects (*n* = 62). From the analysis results, we can see that the six kinds of miRNA statistics show that in the heart failure samples have a significant upward trend. The relative miRNA expression levels were normalized to cel-miR-39 and calculated by -ΔΔCt. Differences between each groups were compared by Kruskal–Wallis ANOVA test; ∗*P* < 0.05.

In order to identify the most stable and reliable candidate miRNAs as the biological target molecule. Based on the literature search, we selected 30 miRNAs as candidate target molecules. Through the preliminary experiments and analysis of the relationship between miRNA expression and heart failure disease, we finally selected six miRNAs, namely, microRNA-21-5p, microRNA-30a-3p, microRNA-30a-5p, microRNA-155-5p, microRNA-216a and microRNA-217. We used internal reference evaluation software RefFinder (http://www.leonxie.com/referencegene.php) to carry out online evaluation of internal reference genes [42], and finally selected the sequence of miR-39 of the nematode as a reference. Related content has been added to the article. RefFinder is a method of assigning weights based on the geometric mean of four commonly used algorithms (geNorm, BestKeeper, NormFinder, and comparativedelta-CT) to rank gene stability [43–46]. We used the prism data analysis software to analyze the data of fluorescence quantitative PCR, and the results were shown in Figure 2. From the analysis results, we can see that the six kinds of miRNA statistics show that in the heart failure samples, the expression of microRNA - 21-5p, microRNA 30a-3p, microRNA 30a-5p, microRNA 155-5p, microRNA 216a and microRNA217 have a significant upward trend. This is also consistent with the conclusions drawn by other research groups, which ensures the accuracy of the experimental results.

### ROC analysis of selected miRNAs

**Figure 3:**
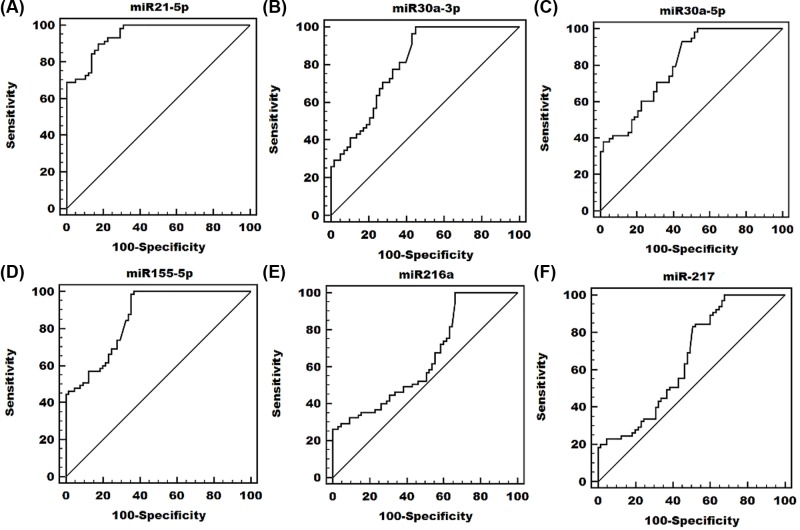
Receiver operating characteristic (ROC) curves analysis of (**A**) miR-21-5p, (**B**) miR-30a-3p, (**C**) miR-30a-5p, (**D**) miR-155-5p, (**E**) miR-216a and (**F**) miR-217 ROC curve evaluation statistics show that all AUC values are greater than 0.5, indicating that the detection method is effective.

We used MedCalc data analysis software to perform ROC analysis of quantitative fluorescent PCR data. The results were shown in Figure 3 and Table 2.

**Table 2:** The areas under the curves (AUC), 95% CI, sensitivity, criterion and specificity of miR-21-5p, miR-30a-3p, miR-30a-5p, miR-155-5p, miR-216a and miR-217

	AUC	95% confidence interval	Sensitivity	Criterion	Specificity
miR−21-5p	0.944	0.886–0.978	89.7	>0.7201	82.8
miR-30a-3p	0.809	0.726–0.876	100.00	>0.9544	55.2
miR-30a-5p	0.801	0.716–0.869	93.1	>0.9988	55.2
miR-155-5p	0.861	0.790–0.916	98.5	>0.8591	64.6
miR-216a	0.648	0.559–0.729	100.00	>0.9178	33.8
miR-217	0.660	0.571–0.740	83.1	>0.9969	49.2

The area under the ROC curve is between 1.0 and 0.5. miR-21-5p, miR-30a-3p, miR-30a-5p, miR-155-5p, miR-216a and miR-217 detection results are accurate.

ROC curve evaluation statistics show that all AUC values are greater than 0.5, indicating that the detection method is effective, and most results greater than 0.7 are more accurate, and the MIC-21-5p AUC value is higher than 0.9 has higher accuracy.

In addition, we carried out a combination test of several miRNAs, and the results of the combination test were shown in the Supplementary Data. From the results of each combination, we can see that the detection results of multiple small RNAs, especially more than three small RNAs, will be more accurate and more reliable.

Similarly, we performed ROC analysis on some of the clinical data collected. The relevant data were listed in the Supplementary Data.

### Correlation analysis

**Figure 4:**
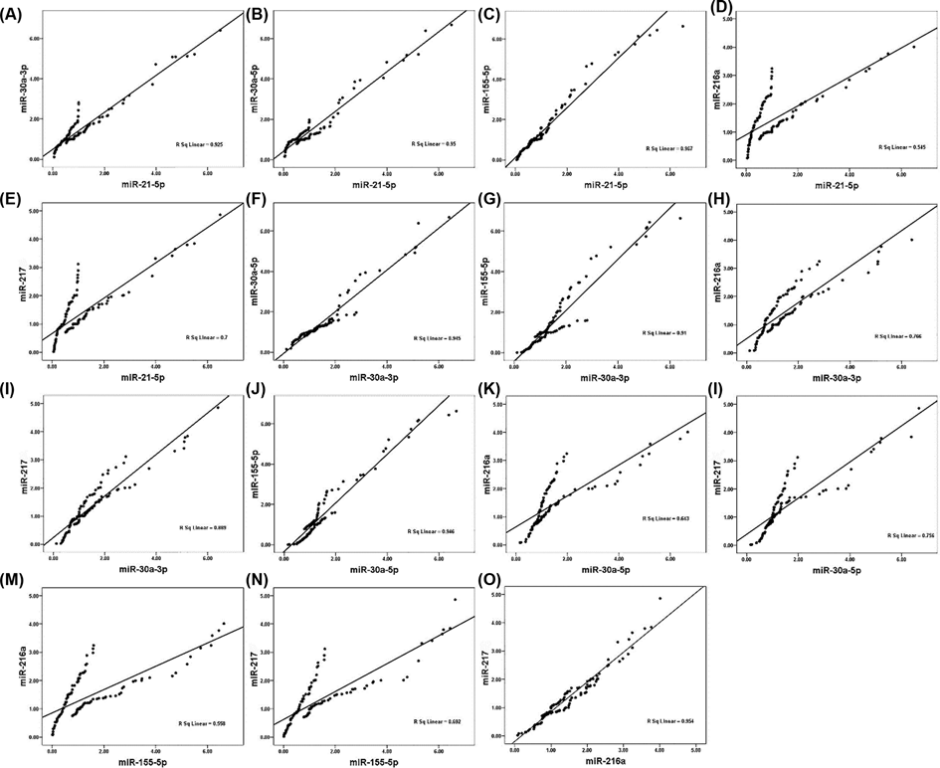
Spearman correlations between the circulating miRNAs in patients with HF The scatter plots show the marked correlation in the expression values between (**A**) miR-21-5p and miR-30a-3p, (**B**) miR-21-5p and miR-30a-5p, (**C**) miR-21-5p and miR-155-5p, (**D**) miR-21-5p and miR-216a, (**E**) miR-21-5p and miR-217, (**F**) miR-30a-3p and miR-30a-5p, (**G**) miR-30a-3p and miR-155-5p, (**H**) miR-30a-3p and miR-216a, (**I**) miR-30a-3p and miR-217, (**J**) miR-30a-5p and miR-155-5p, (**K**) miR-30a-5p and miR-216a, (**L**) miR-30a-5p and miR-217, (**M**) miR-155-5p and miR-216a, (**N**) miR-155-5p and miR-217, (**O**) miR-216a and miR-217, in the population (*n* = 62) with AMI.

We used SPSS analysis software to analyze the correlation of the quantitative data. The results were shown in Figure 4 and Table 3.

**Table 3:** Spearman correlations between the circulating miRNAs in patients with HF, list of *P* and *r* result

		miR−21-5p	miR-30a-3p	miR-30a-5p	miR-155-5p	miR-216a-5p	miR-217-5p
miR−21-5p	*r*		0.976	0.987	0.983	0.738	0.836
	*P*		0.000	0.000	0.000	0.000	0.000
miR-30a-3p	*P*	0.976		0.981	0.954	0.854	0.943
	sig	0.000		0.000	0.000	0.000	0.000
miR-30a-5p	*P*	0.987	0.981		0.973	0.802	0.870
	sig	0.000	0.000		0.000	0.000	0.000
miR-155-5p	*P*	0.983	0.954	0.973		0.747	0.832
	sig	0.000	0.000	0.000		0.000	0.000
miR-216a	*P*	0.738	0.875	0.802	0.747		0.977
	sig	0.000	0.000	0.000	0.000		0.000
miR-217	*P*	0.836	0.943	0.870	0.832	0.977	
	sig	0.000	0.000	0.000	0.000	0.000	

From the experimental results, we can see that there is a significant correlation between the six small RNAs, which proves that the results are related. The consistency of test results is ensured.

Similarly, we analyzed the correlation between the miRNA test results and the clinical gold standard test results. The results were shown in Supplementary Data.

## Summary

Up to now, many studies have shown that miRNAs as biomarkers have important clinical applications, but it is still necessary to study whether miRNAs can establish reliable diagnostic and potential biomarkers for heart failure to verify their accuracy and repeatability. We found that plasma levels of miR-21-5p, miR-30a-3p, miR-30a-5p, miR-155-5p, miR-216a and miR-217 were not affected by hemolysis, age, and gender when used to diagnose heart failure. miR-21-5p, miR-30a-3p, miR-30a-5p, miR-155-5p, miR-216a and miR-217 may be a new biomarker for the diagnosis of heart failure and related diseases. These microRNAs have potential as diagnostic and prognostic biomarkers and will be used in clinical diagnosis and treatment of heart failure diseases in conjunction with existing gold standards.

## Supplementary Material

Supplementary Figures S1-S29 and Tables S1-S29

## Abbreviations

CT: cyclic threshold
miRNA: microRNA
qRT-PCR: quantitative real-time polymerase chain reaction
