# Supplementary material for: Combined detection of miR-21-5p, miR-30a-3p, miR-30a-5p, miR-155-5p, miR-216a and miR-217 for screening of early heart failure diseases
Source: Biosci Rep. 2020 Mar 18;40(3):BSR20191653. doi: 10.1042/BSR20191653 (PMC7080642; doi:10.1042/BSR20191653)
Supplement: Supplementary Figures S1-S29 and Tables S1-S29 [file BSR-2019-1653_supp.pdf]

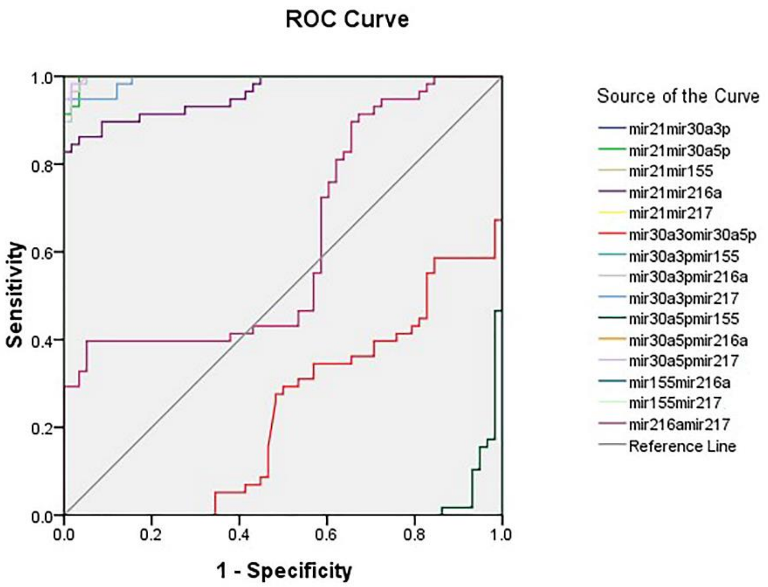

Fig. S1. Receiver operating characteristic (ROC) curves analysis of pairwise covering.

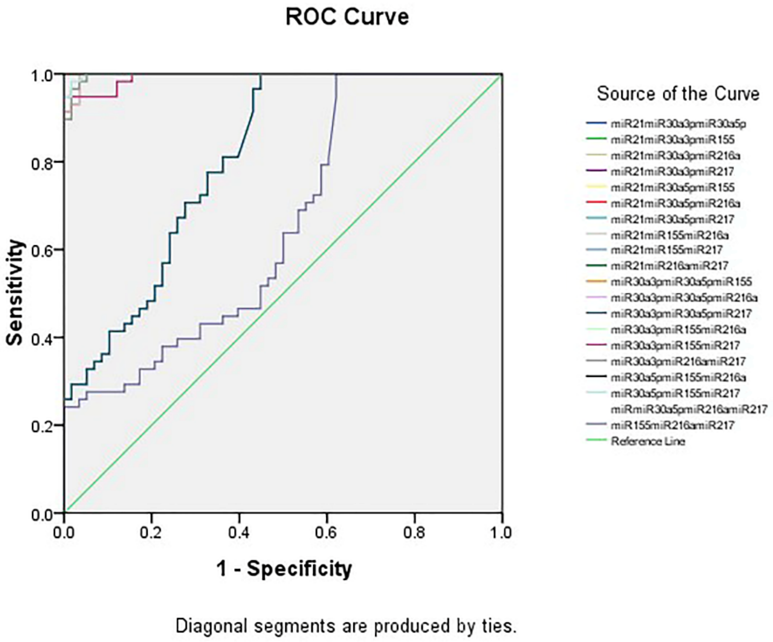

Fig. S2. Receiver operating characteristic (ROC) curves analysis of three combinations.

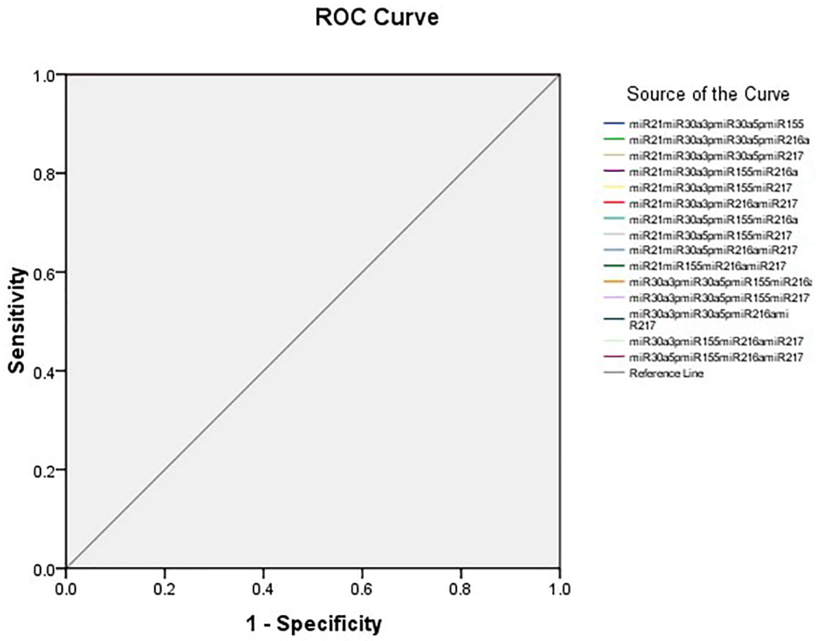

Fig. S3. Receiver operating characteristic (ROC) curves analysis of four combinations.

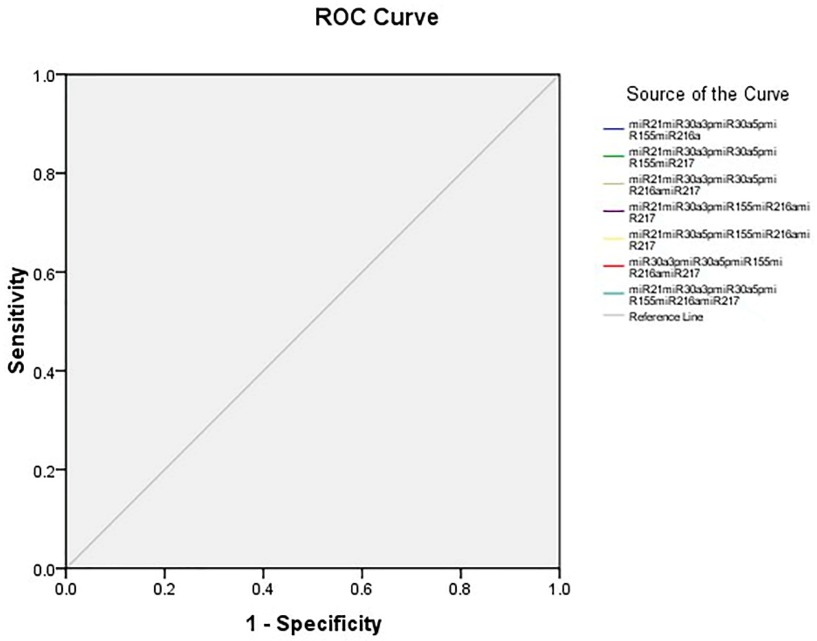

Fig. S4. Receiver operating characteristic (ROC) curves analysis of five combinations.

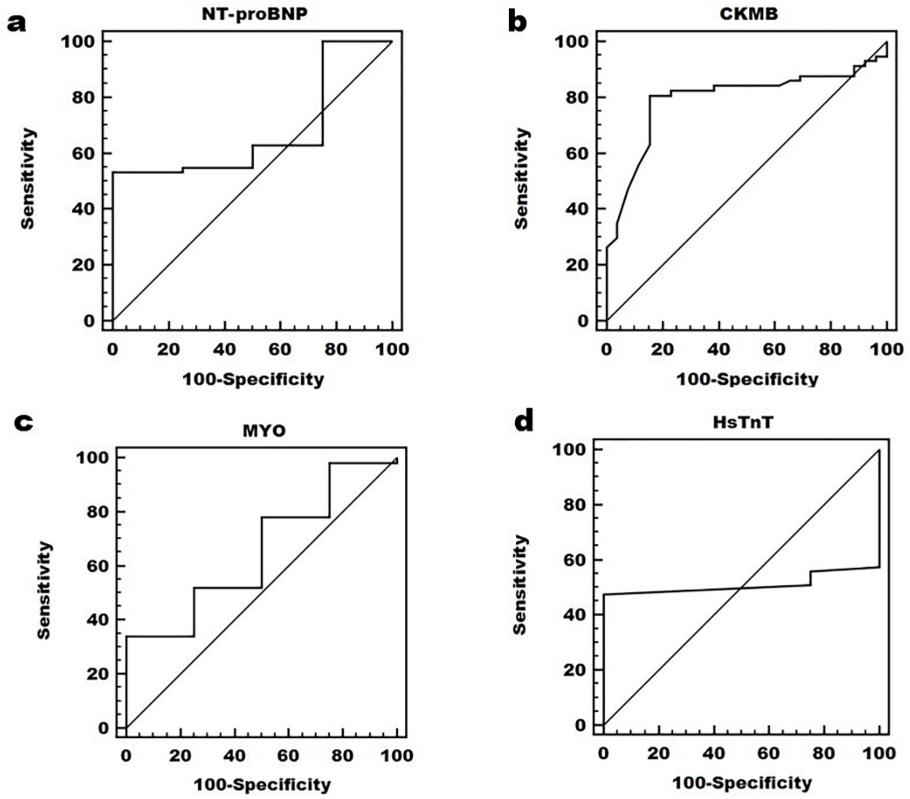

Fig. S5. Receiver operating characteristic (ROC) curves analysis of clinical laboratory indicators, (a) NT-proBNP, (b) CKMB, (c) MYO and (d) HsTnT.

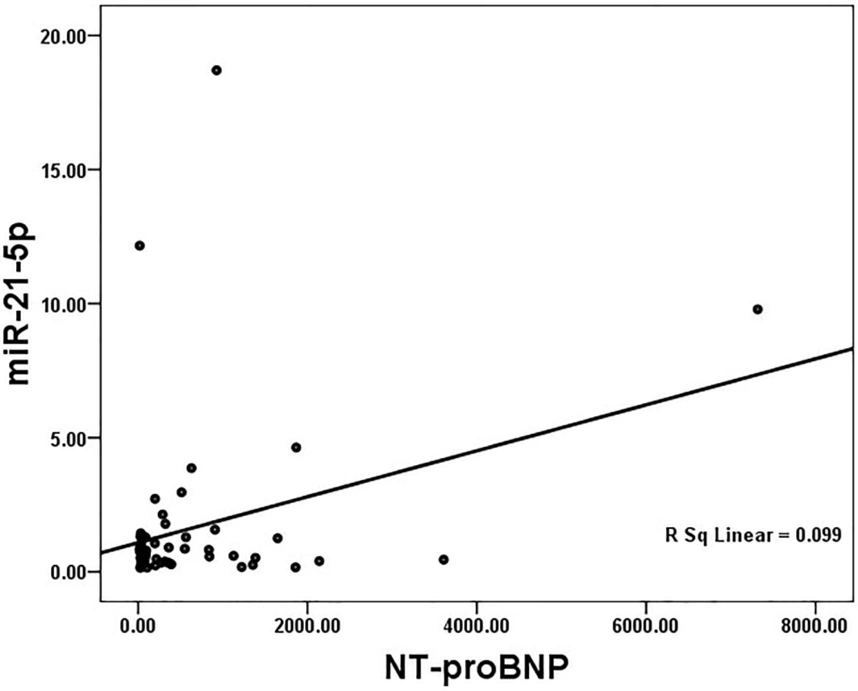

Fig S6. Spearman correlations between the circulating miRNAs in patients with HF. The scatter plots show the marked correlation in the expression values between miR-21-5p and NT-proBNP.

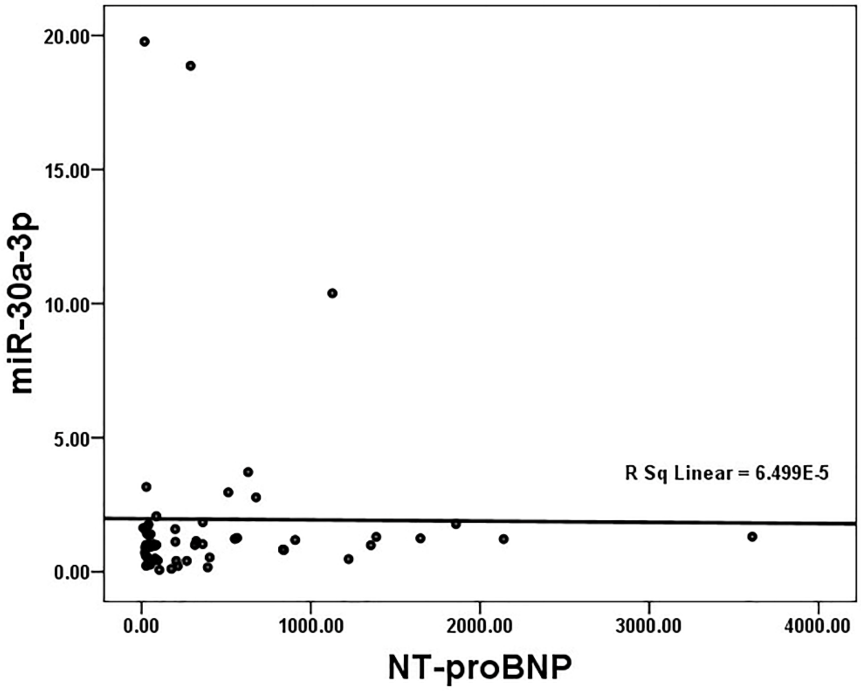

Fig S7. Spearman correlations between the circulating miRNAs in patients with HF. The scatter plots show the marked correlation in the expression values between miR-30a-3p and NT-proBNP.

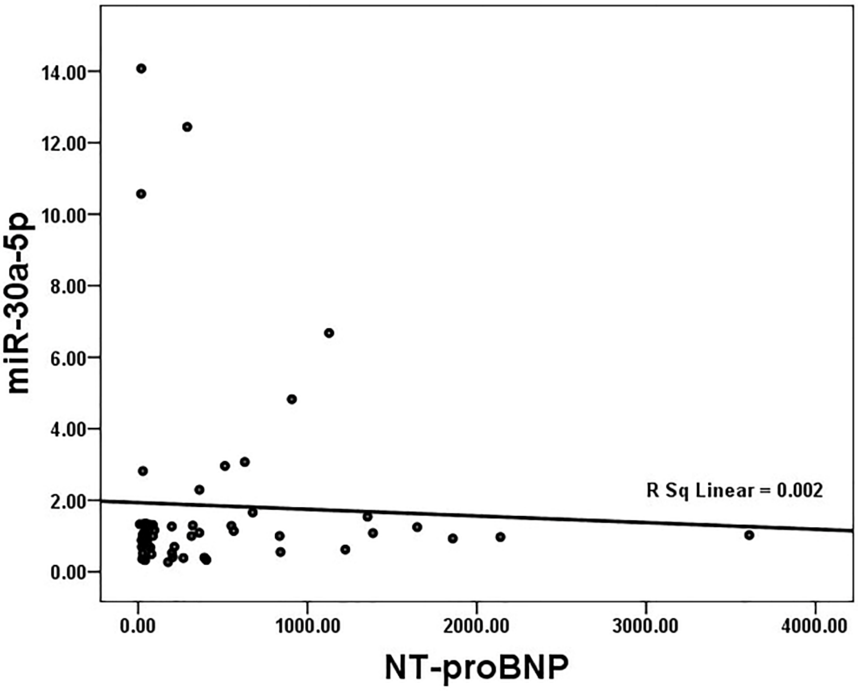

Fig S8. Spearman correlations between the circulating miRNAs in patients with HF. The scatter plots show the marked correlation in the expression values between miR-30a-5p and NT-proBNP.

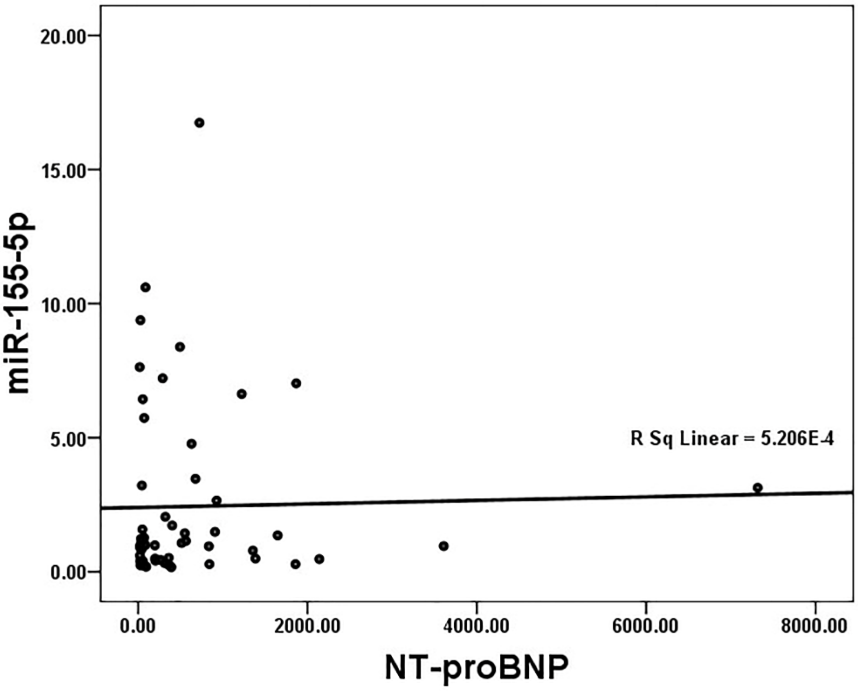

Fig S9. Spearman correlations between the circulating miRNAs in patients with HF. The scatter plots show the marked correlation in the expression values between miR-155-5p and NT-proBNP.

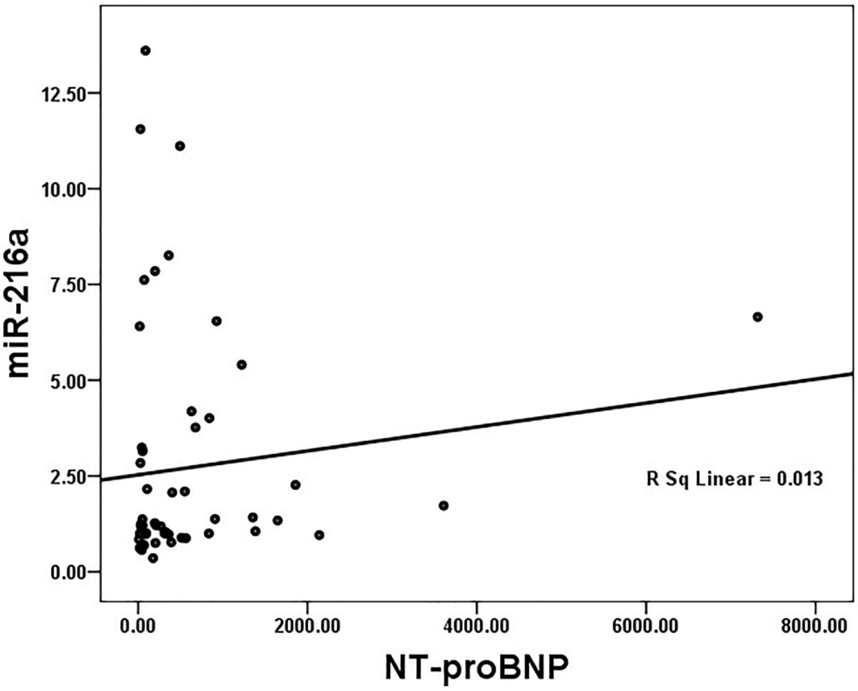

Fig S10. Spearman correlations between the circulating miRNAs in patients with HF. The scatter plots show the marked correlation in the expression values between miR-216a and NT-proBNP.

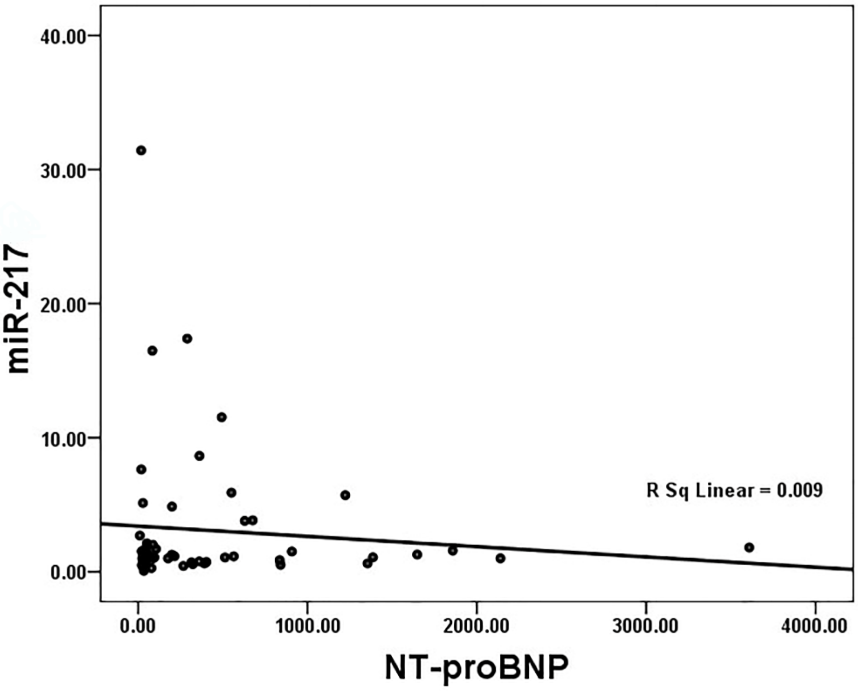

Fig S11. Spearman correlations between the circulating miRNAs in patients with HF. The scatter plots show the marked correlation in the expression values between miR-217 and NT-proBNP.

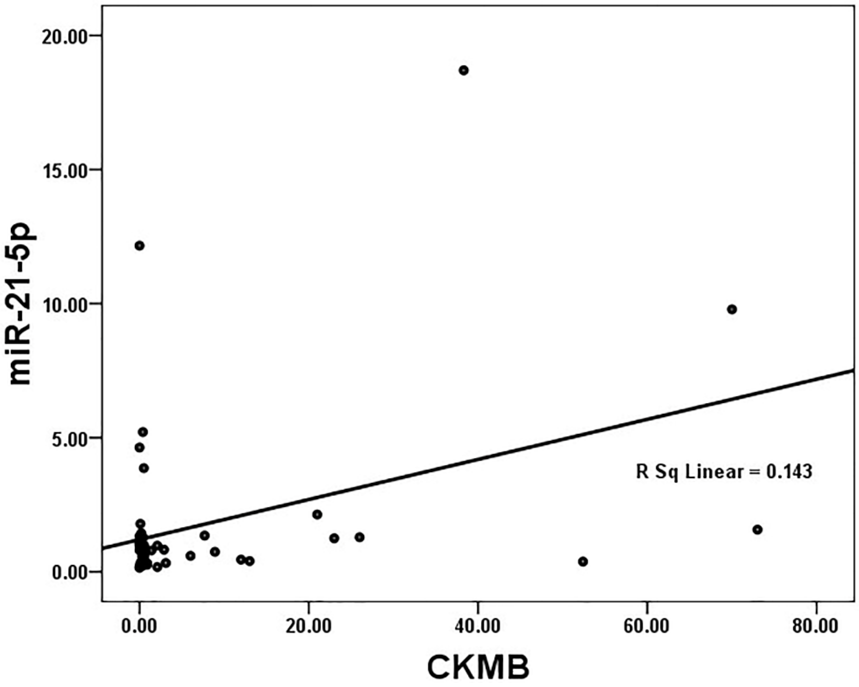

Fig S12. Spearman correlations between the circulating miRNAs in patients with HF. The scatter plots show the marked correlation in the expression values between miR-21-5p and CKMB.

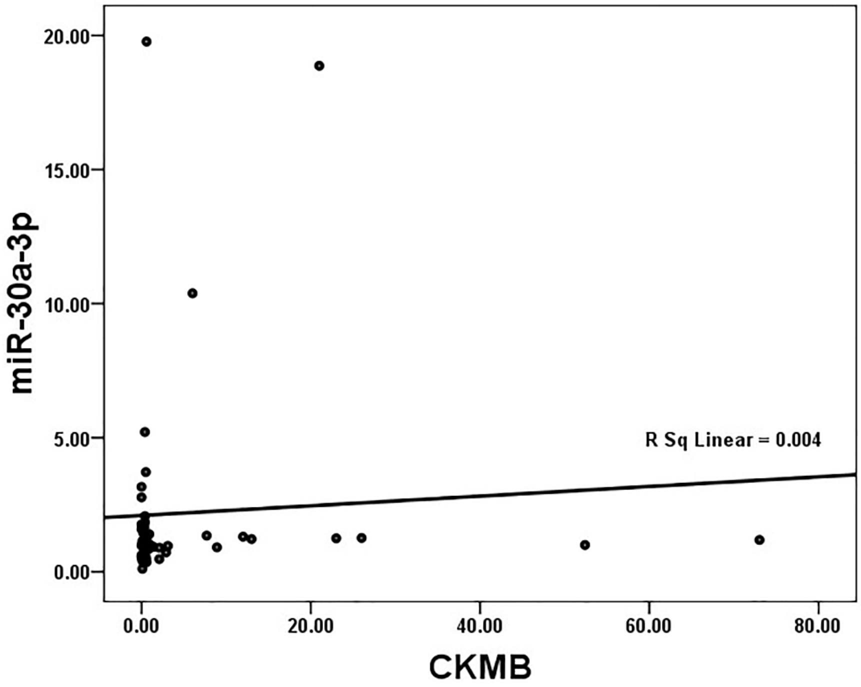

Fig S13. Spearman correlations between the circulating miRNAs in patients with HF. The scatter plots show the marked correlation in the expression values between miR-30a-3p and CKMB.

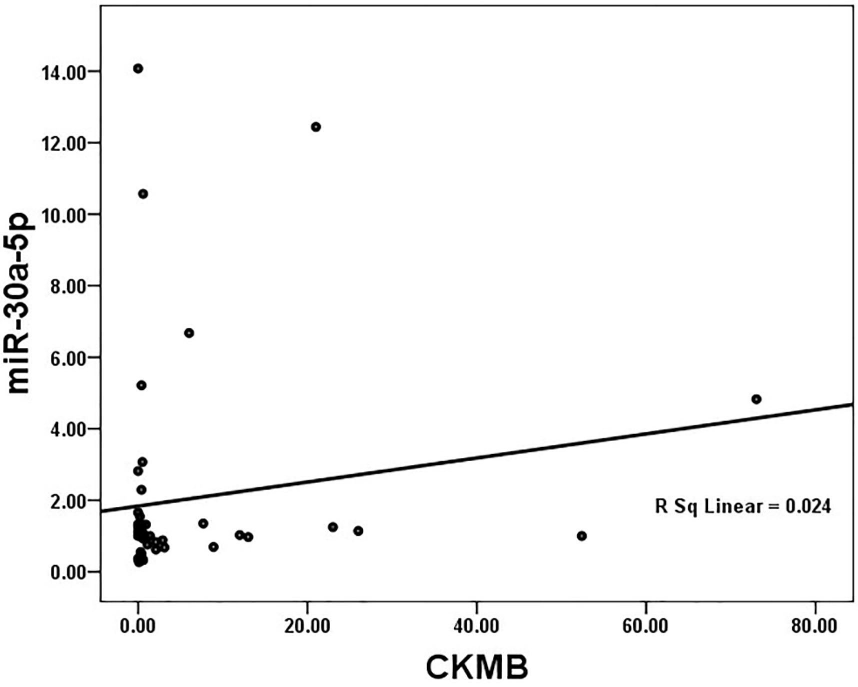

Fig S14. Spearman correlations between the circulating miRNAs in patients with HF. The scatter plots show the marked correlation in the expression values between miR-30a-5p and CKMB.

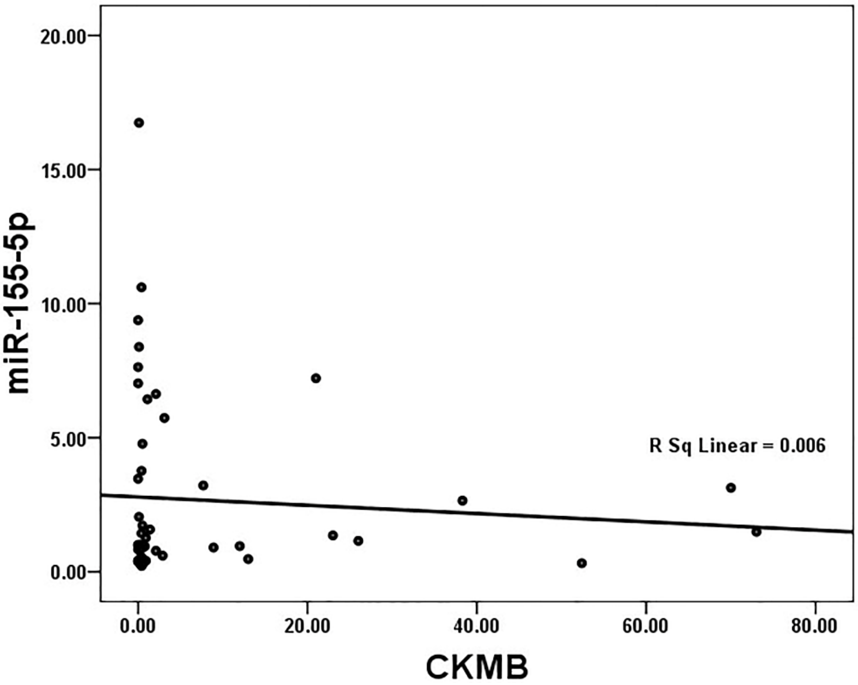

Fig S15. Spearman correlations between the circulating miRNAs in patients with HF. The scatter plots show the marked correlation in the expression values between miR-155-5p and CKMB.

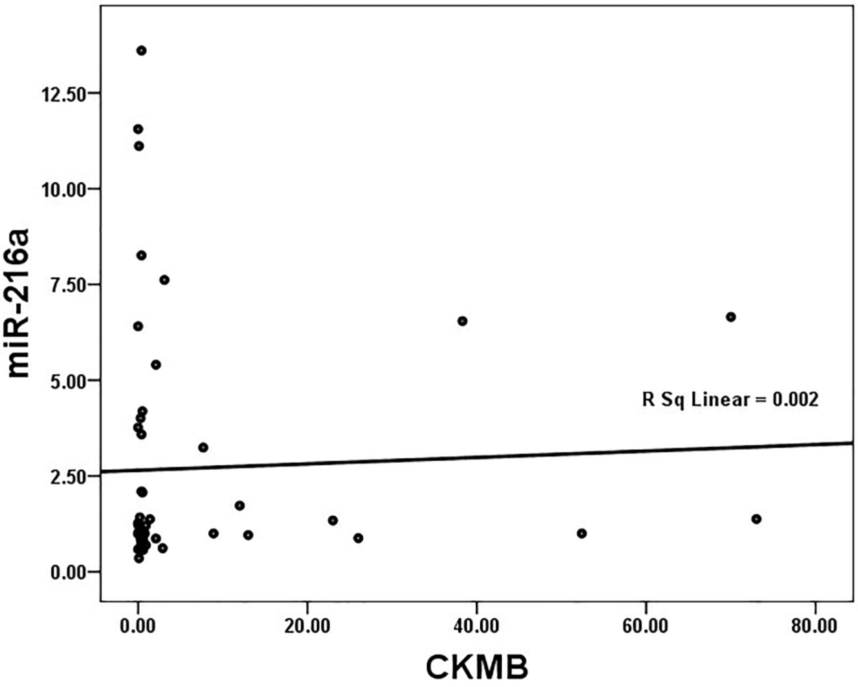

Fig S16. Spearman correlations between the circulating miRNAs in patients with HF. The scatter plots show the marked correlation in the expression values between miR-216a and CKMB.

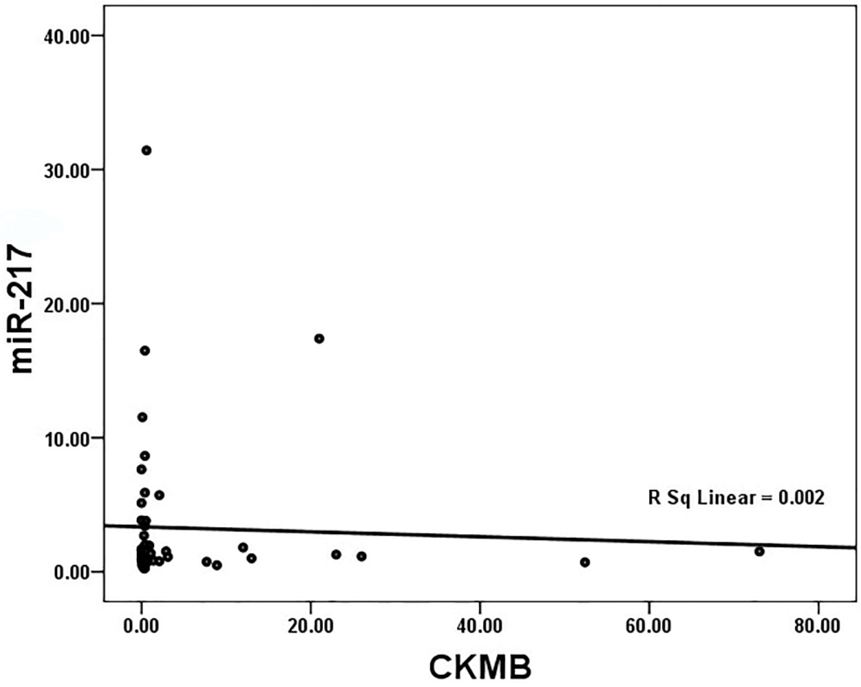

Fig S17. Spearman correlations between the circulating miRNAs in patients with HF. The scatter plots show the marked correlation in the expression values between miR-217 and CKMB.

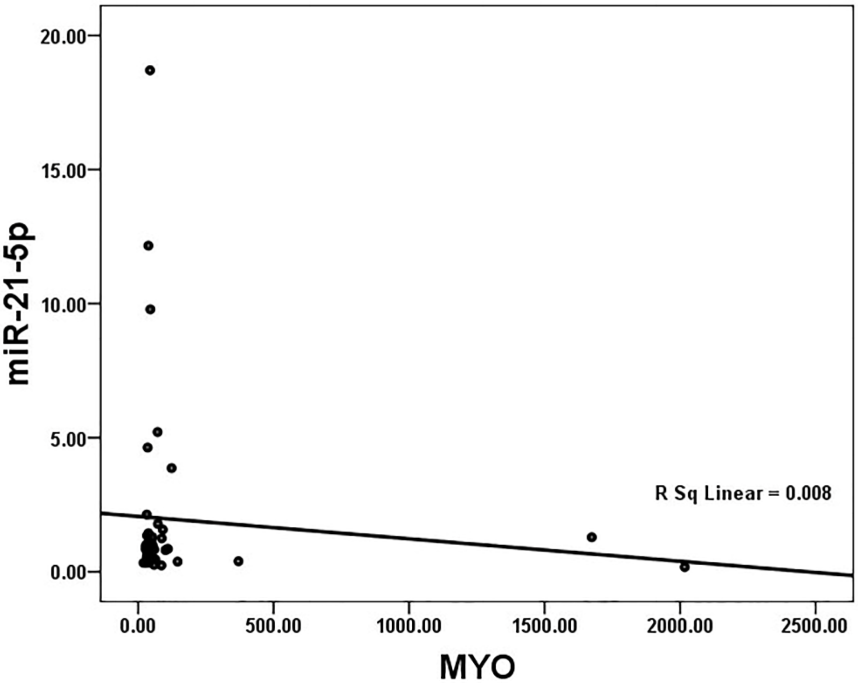

Fig S18. Spearman correlations between the circulating miRNAs in patients with HF. The scatter plots show the marked correlation in the expression values between miR-21-5p and MYO.

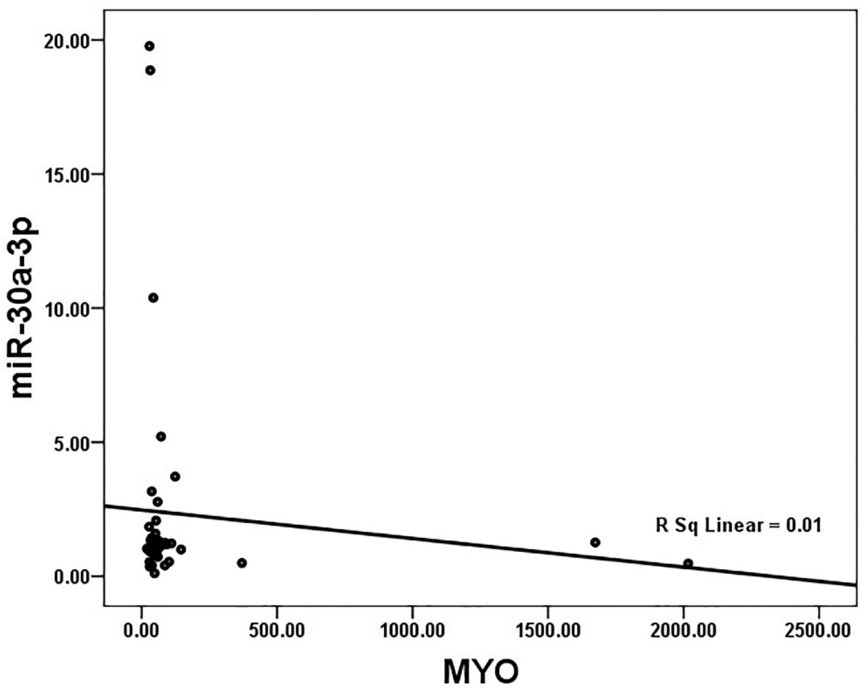

Fig S19. Spearman correlations between the circulating miRNAs in patients with HF. The scatter plots show the marked correlation in the expression values between miR-30a-3p and MYO.

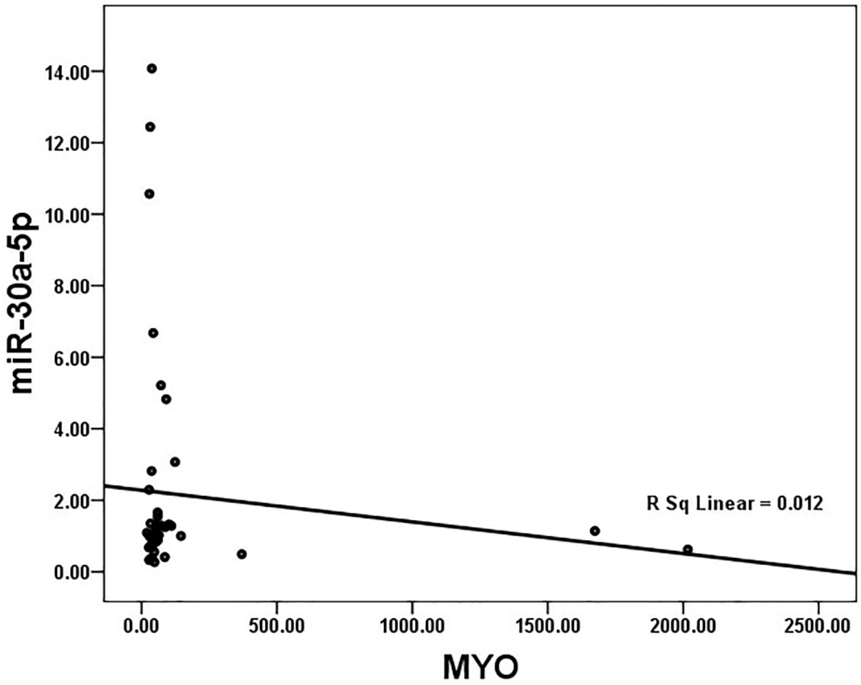

Fig S20. Spearman correlations between the circulating miRNAs in patients with HF. The scatter plots show the marked correlation in the expression values between miR-30a-5p and MYO.

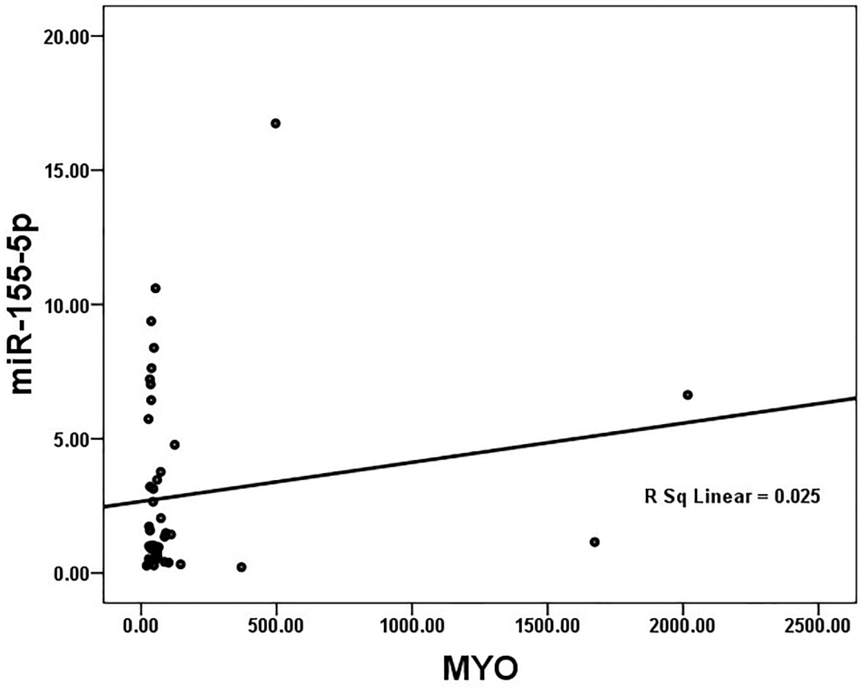

Fig S21. Spearman correlations between the circulating miRNAs in patients with HF. The scatter plots show the marked correlation in the expression values between miR-155-5p and MYO.

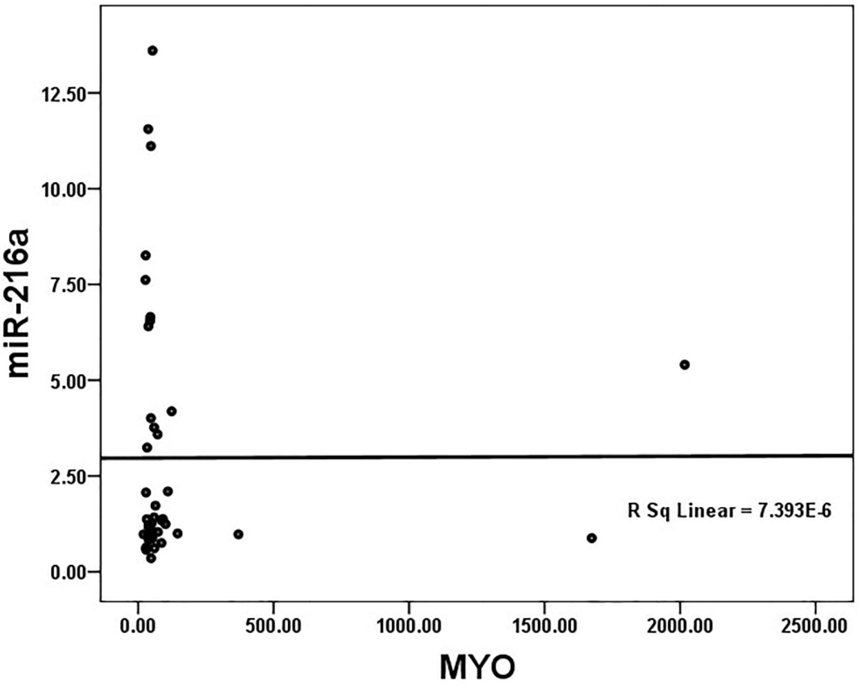

Fig S22. Spearman correlations between the circulating miRNAs in patients with HF. The scatter plots show the marked correlation in the expression values between miR-216a and MYO.

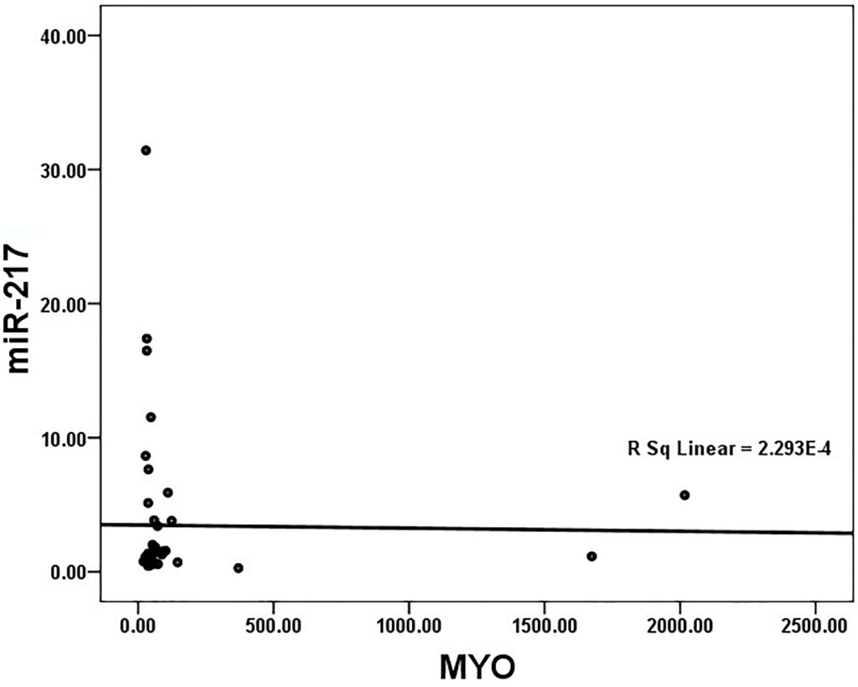

Fig S23. Spearman correlations between the circulating miRNAs in patients with HF. The scatter plots show the marked correlation in the expression values between miR-217 and MYO.

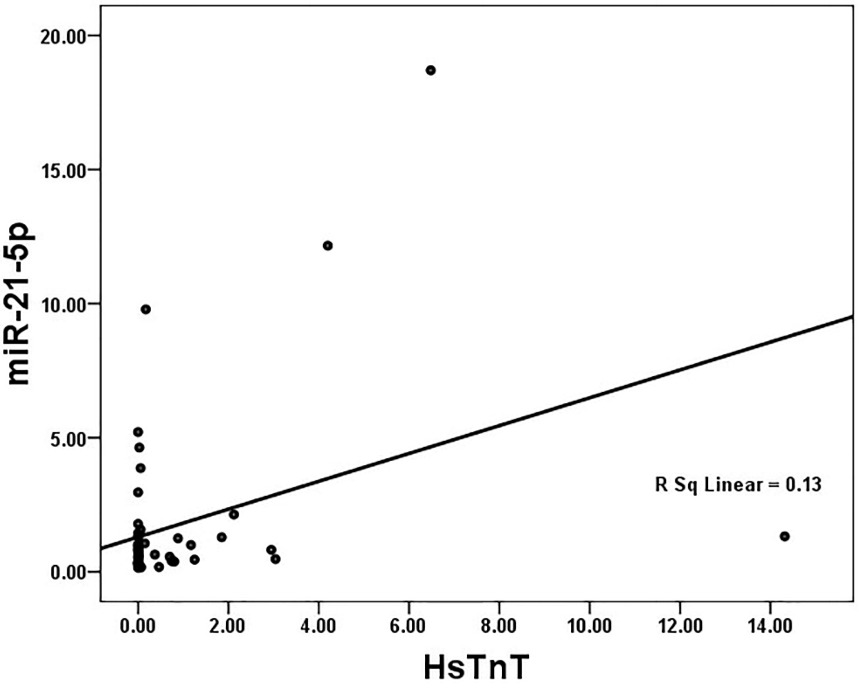

Fig S24. Spearman correlations between the circulating miRNAs in patients with HF. The scatter plots show the marked correlation in the expression values between miR-21-5p and HsTnT.

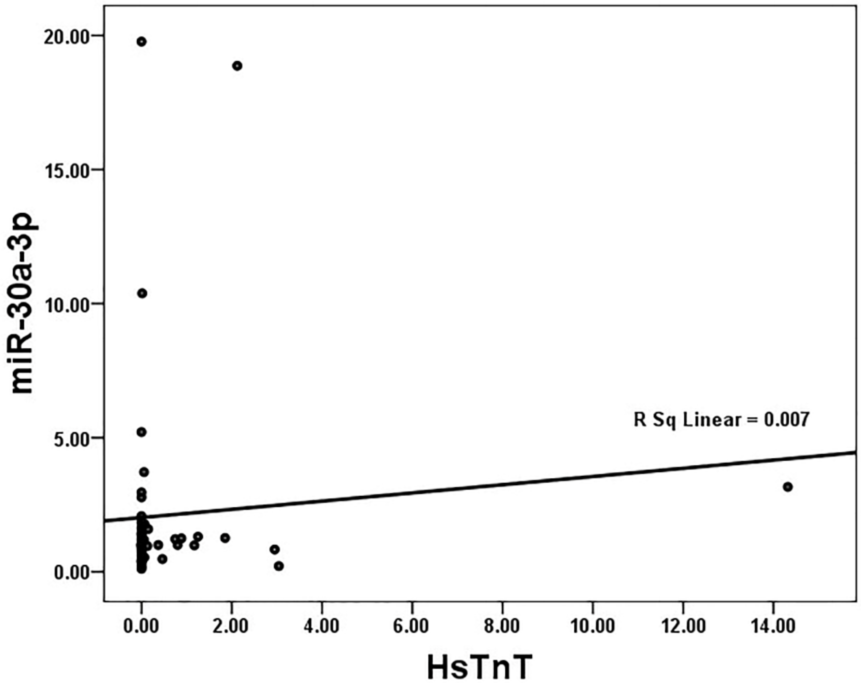

Fig S25. Spearman correlations between the circulating miRNAs in patients with HF. The scatter plots show the marked correlation in the expression values between miR-30a-3p and HsTnT.

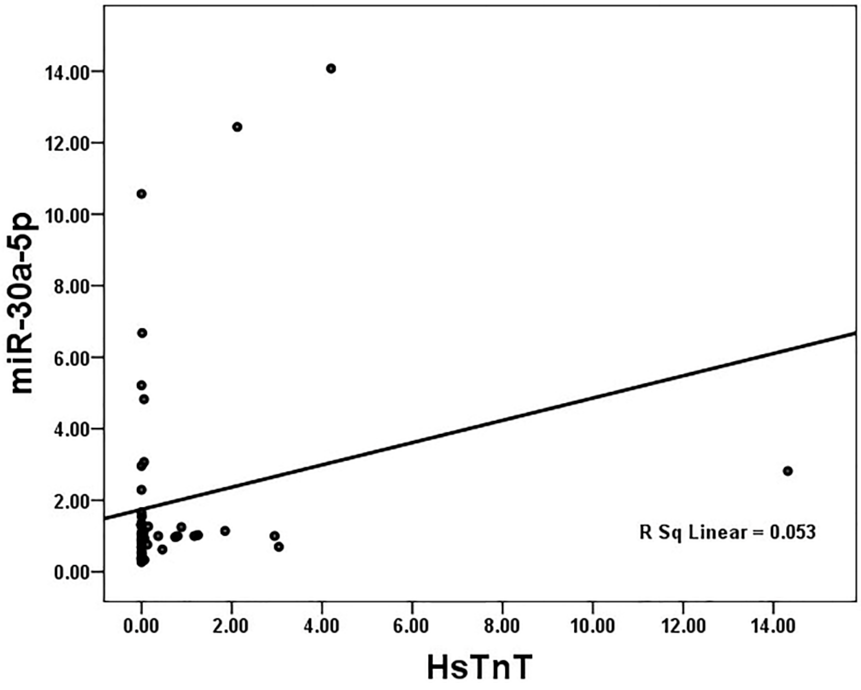

Fig S26. Spearman correlations between the circulating miRNAs in patients with HF. The scatter plots show the marked correlation in the expression values between miR-30a-5p and HsTnT.

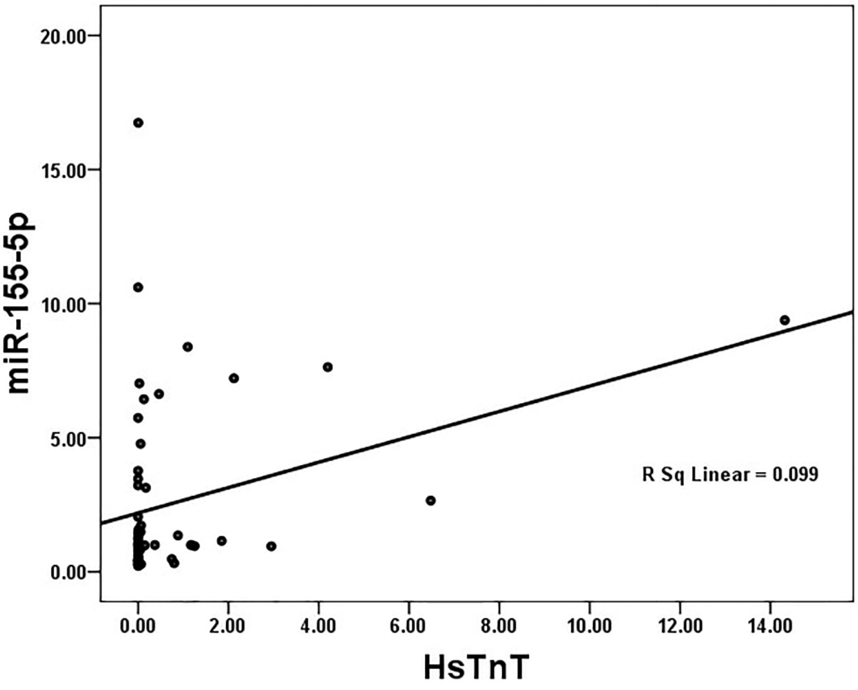

Fig S27. Spearman correlations between the circulating miRNAs in patients with HF. The scatter plots show the marked correlation in the expression values between miR-155-5p and HsTnT.

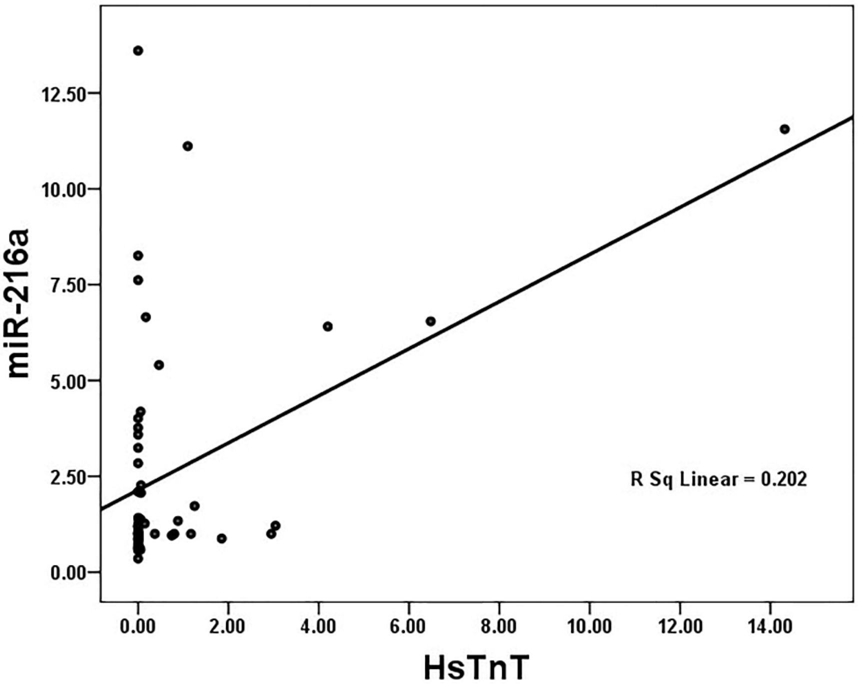

Fig S28. Spearman correlations between the circulating miRNAs in patients with HF. The scatter plots show the marked correlation in the expression values between miR-216a and HsTnT.

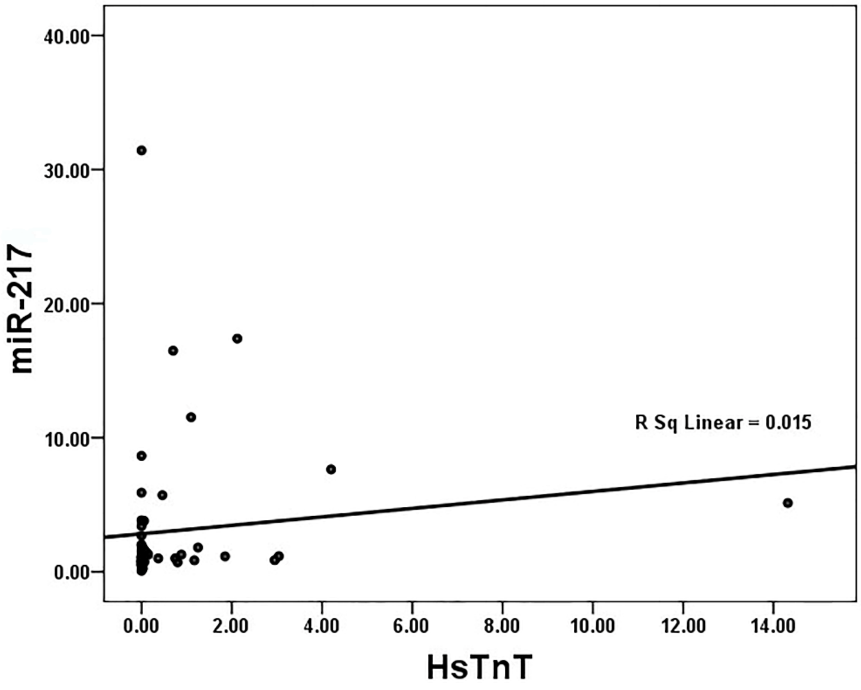

Fig S29. Spearman correlations between the circulating miRNAs in patients with HF. The scatter plots show the marked correlation in the expression values between miR-217 and HsTnT.

Table S1 primers used for RT-PCR

microRNA	Primers sequences
miR-21-5p	GGACACCTAGCTTATCAGACTGATGTTGA
miR-30a-3p	GGACCCTTTCAGTCGGATGTTTG
miR-30a-5p	GGACCTGTAAACATCCTCGACTG
miR-155-5p	GGACCTTAATGCTAATCGTGATAGGGGT
miR-216a	GGACTAATCTCAGCTGGCAACTGTGA
miR-217	GGACTACTGCATCAGGAACTGATTGGA
Cel-miR-39	TCACCGGGTGTAAATCAGCTTG

Table. S1. The areas under the curves (AUC), 95% CI, Sensitivity, Criterion and Specificity of pairwise covering.

Area Under the Curve

Test Result Variable(s)	Area	Std. Error[a]	Asymptotic Sig.[b]	Asymptotic 95% Confidence Interval	
				Lower Bound	Upper Bound
mir21mir30a3p	1.000	.000	.000	1.000	1.000
mir21mir30a5p	.997	.002	.000	.993	1.002
mir21mir155	1.000	.000	.000	1.000	1.000
mir21mir216a	.960	.016	.000	.927	.992
mir21mir217	1.000	.000	.000	1.000	1.000
mir30a3omir30a5p	.237	.043	.000	.152	.322
mir30a3pmir155	.000	.000	.000	.000	.000
mir30a3pmir216a	.997	.002	.000	.993	1.002
mir30a3pmir217	.993	.005	.000	.984	1.002
mir30a5pmir155	.017	.010	.000	-.002	.036
mir30a5pmir216a	1.000	.000	.000	1.000	1.000
mir30a5pmir217	.999	.001	.000	.996	1.001
mir155mir216a	1.000	.000	.000	1.000	1.000
mir155mir217	1.000	.000	.000	1.000	1.000
mir216amir217	.625	.053	.021	.520	.729

The test result variable(s): mir30a3omir30a5p, mir30a5pmir217 has at least one tie between the positive actual state group and the negative actual state group. Statistics may be biased.

a. Under the nonparametric assumption

b. Null hypothesis: true area = 0.5

Table. S2. The areas under the curves (AUC), 95% CI, Sensitivity, Criterion and Specificity of three combinations.

Area Under the Curve

Test Result Variable(s)	Area	Std. Error[a]	Asymptotic Sig.[b]	Asymptotic 95% Confidence Interval	
				Lower Bound	Upper Bound
miR21miR30a3pmiR30a5p	.809	.039	.000	.732	.887
miR21miR30a3pmiR155	1.000	.000	.000	1.000	1.000
miR21miR30a3pmiR216a	.997	.002	.000	.993	1.002
miR21miR30a3pmiR217	1.000	.000	.000	1.000	1.000
miR21miR30a5pmiR155	.809	.039	.000	.732	.887
miR21miR30a5pmiR216a	.997	.002	.000	.993	1.002
miR21miR30a5pmiR217	1.000	.000	.000	1.000	1.000
miR21miR155miR216a	.997	.002	.000	.993	1.002
miR21miR155miR217	1.000	.000	.000	1.000	1.000
miR21miR216amiR217	1.000	.000	.000	1.000	1.000
miR30a3pmiR30a5pmiR155	1.000	.000	.000	1.000	1.000
miR30a3pmiR30a5pmiR216a	.809	.039	.000	.732	.887
miR30a3pmiR30a5pmiR217	.809	.039	.000	.732	.887
miR30a3pmiR155miR216a	.997	.002	.000	.993	1.002
miR30a3pmiR155miR217	.993	.005	.000	.984	1.002
miR30a3pmiR216amiR217	.997	.002	.000	.993	1.002
miR30a5pmiR155miR216a	1.000	.000	.000	1.000	1.000
miR30a5pmiR155miR217	.999	.001	.000	.996	1.001
miRmiR30a5pmiR216amiR217	1.000	.000	.000	1.000	1.000
miR155miR216amiR217	.654	.051	.004	.554	.754

The test result variable(s): miR21miR30a3pmiR30a5p, miR21miR30a5pmiR155, miR30a3pmiR30a5pmiR216a, miR30a3pmiR30a5pmiR217, miR30a5pmiR155miR217, miR155miR216amiR217 has at least one tie between the positive actual state group and the negative actual state group. Statistics may be biased.

a. Under the nonparametric assumption

b. Null hypothesis: true area = 0.5

Table. S3. The areas under the curves (AUC), 95% CI, Sensitivity, Criterion and Specificity of four combinations.

Area Under the Curve

Test Result Variable(s)	Area	Std. Error[a]	Asymptotic Sig.[b]	Asymptotic 95% Confidence Interval	
				Lower Bound	Upper Bound
miR21miR30a3pmiR30a5pmiR155	1.000	.000	.000	1.000	1.000
miR21miR30a3pmiR30a5pmiR216a	1.000	.000	.000	1.000	1.000
miR21miR30a3pmiR30a5pmiR217	1.000	.000	.000	1.000	1.000
miR21miR30a3pmiR155miR216a	1.000	.000	.000	1.000	1.000
miR21miR30a3pmiR155miR217	1.000	.000	.000	1.000	1.000
miR21miR30a3pmiR216amiR217	1.000	.000	.000	1.000	1.000
miR21miR30a5pmiR155miR216a	1.000	.000	.000	1.000	1.000
miR21miR30a5pmiR155miR217	1.000	.000	.000	1.000	1.000
miR21miR30a5pmiR216amiR217	1.000	.000	.000	1.000	1.000
miR21miR155miR216amiR217	1.000	.000	.000	1.000	1.000
miR30a3pmiR30a5pmiR155miR216a	1.000	.000	.000	1.000	1.000
miR30a3pmiR30a5pmiR155miR217	1.000	.000	.000	1.000	1.000
miR30a3pmiR30a5pmiR216amiR217	1.000	.000	.000	1.000	1.000
miR30a3pmiR155miR216amiR217	1.000	.000	.000	1.000	1.000
miR30a5pmiR155miR216amiR217	1.000	.000	.000	1.000	1.000

a. Under the nonparametric assumption

b. Null hypothesis: true area = 0.5

Table. S4. The areas under the curves (AUC), 95% CI, Sensitivity, Criterion and Specificity of five combinations.

Area Under the Curve

Test Result Variable(s)	Area	Std. Error[a]	Asymptotic Sig.[b]	Asymptotic 95% Confidence Interval	
				Lower Bound	Upper Bound
miR21miR30a3pmiR30a5pmiR155miR216a	1.000	.000	.000	1.000	1.000
miR21miR30a3pmiR30a5pmiR155miR217	1.000	.000	.000	1.000	1.000
miR21miR30a3pmiR30a5pmiR216amiR217	1.000	.000	.000	1.000	1.000
miR21miR30a3pmiR155miR216amiR217r	1.000	.000	.000	1.000	1.000
miR21miR30a5pmiR155miR216amiR217	1.000	.000	.000	1.000	1.000
miR30a3pmiR30a5pmiR155miR216amiR217	1.000	.000	.000	1.000	1.000
miR21miR30a3pmiR30a5pmiR155miR216amiR217	1.000	.000	.000	1.000	1.000

a. Under the nonparametric assumption

b. Null hypothesis: true area = 0.5

Table. S5. The areas under the curves (AUC), 95% CI, Sensitivity, Criterion and Specificity of clinical laboratory indicators: NT-proBNP, CKMB, MYO and HsTnT.

	AUC	95% confidence Interval	Sensitivity	Criterion	Specificity
NT-proBNP	0.677	0.551 to 0.787	53.2	>197.8	100.0
CKMB	0.795	0.692 to 0.875	80.7	≤6.02	84.6
MYO	0.655	0.513 to 0.779	34.0	>55.6	100.0
HsTnT	0.510	0.383 to 0.636	47.5	≤0.0016	100.0

Table S6. Spearman correlations between miR-21-5p and NT-proBNP in patients with HF, list of p and sig result.

Correlations

		mir21	NTproBNP
mir21	Pearson Correlation	1	.314*
	Sig. (2-tailed)		.020
	N	59	55
NTproBNP	Pearson Correlation	.314*	1
	Sig. (2-tailed)	.020	
	N	55	62

*. Correlation is significant at the 0.05 level (2-tailed).

Table S7. Spearman correlations between miR-30a-3p and NT-proBNP in patients with HF, list of p and sig result.

Correlations

		mir30a3p	NTproBNP
mir30a3p	Pearson Correlation	1	-.008
	Sig. (2-tailed)		.953
	N	59	55
NTproBNP	Pearson Correlation	-.008	1
	Sig. (2-tailed)	.953	
	N	55	62

Table S8. Spearman correlations between miR-30a-5p and NT-proBNP in patients with HF, list of p and sig result.

Correlations

		mir30a5p	NTproBNP
mir30a5p	Pearson Correlation	1	-.045
	Sig. (2-tailed)		.745
	N	59	55
NTproBNP	Pearson Correlation	-.045	1
	Sig. (2-tailed)	.745	
	N	55	62

Table S9. Spearman correlations between miR-155-5p and NT-proBNP in patients with HF, list of p and sig result.

Correlations

		mir155	NTproBNP
mir155	Pearson Correlation	1	.023
	Sig. (2-tailed)		.869
	N	59	55
NTproBNP	Pearson Correlation	.023	1
	Sig. (2-tailed)	.869	
	N	55	62

Table S10. Spearman correlations between miR-216a and NT-proBNP in patients with HF, list of p and sig result.

Correlations

		mir216a	NTproBNP
mir216a	Pearson Correlation	1	.115
	Sig. (2-tailed)		.402
	N	59	55
NTproBNP	Pearson Correlation	.115	1
	Sig. (2-tailed)	.402	
	N	55	62

Table S11. Spearman correlations between miR-217 and NT-proBNP in patients with HF, list of p and sig result.

Correlations

		mir217	NTproBNP
mir217	Pearson Correlation	1	-.096
	Sig. (2-tailed)		.485
	N	59	55
NTproBNP	Pearson Correlation	-.096	1
	Sig. (2-tailed)	.485	
	N	55	62

Table S12. Spearman correlations between miR-21-5p and CKMB in patients with HF, list of p and sig result.

Correlations

		mir21	CKMB
mir21	Pearson Correlation	1	.378**
	Sig. (2-tailed)		.007
	N	59	49
CKMB	Pearson Correlation	.378**	1
	Sig. (2-tailed)	.007	
	N	49	56

**. Correlation is significant at the 0.01 level (2-tailed).

Table S13. Spearman correlations between miR-30a-3p and CKMB in patients with HF, list of p and sig result.

Correlations

		mir30a3p	CKMB
mir30a3p	Pearson Correlation	1	.062
	Sig. (2-tailed)		.670
	N	59	49
CKMB	Pearson Correlation	.062	1
	Sig. (2-tailed)	.670	
	N	49	56

Table S14. Spearman correlations between miR-30a-5p and CKMB in patients with HF, list of p and sig result.

Correlations

		mir30a5p	CKMB
mir30a5p	Pearson Correlation	1	.154
	Sig. (2-tailed)		.285
	N	59	50
CKMB	Pearson Correlation	.154	1
	Sig. (2-tailed)	.285	
	N	50	56

Table S15. Spearman correlations between miR-155-5p and CKMB in patients with HF, list of p and sig result.

Correlations

		mir155	CKMB
mir155	Pearson Correlation	1	-.076
	Sig. (2-tailed)		.595
	N	59	51
CKMB	Pearson Correlation	-.076	1
	Sig. (2-tailed)	.595	
	N	51	56

Table S16. Spearman correlations between miR-216a and CKMB in patients with HF, list of p and sig result.

Correlations

		mir216a	CKMB
mir216a	Pearson Correlation	1	.045
	Sig. (2-tailed)		.760
	N	59	49
CKMB	Pearson Correlation	.045	1
	Sig. (2-tailed)	.760	
	N	49	56

Table S17. Spearman correlations between miR-217 and CKMB in patients with HF, list of p and sig result.

Correlations

		mir217	CKMB
mir217	Pearson Correlation	1	-.045
	Sig. (2-tailed)		.759
	N	59	49
CKMB	Pearson Correlation	-.045	1
	Sig. (2-tailed)	.759	
	N	49	56

Table S18. Spearman correlations between miR-21-5p and MYO in patients with HF, list of p and sig result.

Correlations

		mir21	MYO
mir21	Pearson Correlation	1	-.091
	Sig. (2-tailed)		.555
	N	59	44
MYO	Pearson Correlation	-.091	1
	Sig. (2-tailed)	.555	
	N	44	50

Table S19. Spearman correlations between miR-30a-3p and MYO in patients with HF, list of p and sig result.

Correlations

		mir30a3p	MYO
mir30a3p	Pearson Correlation	1	-.099
	Sig. (2-tailed)		.528
	N	59	43
MYO	Pearson Correlation	-.099	1
	Sig. (2-tailed)	.528	
	N	43	50

Table S20. Spearman correlations between miR-30a-5p and MYO in patients with HF, list of p and sig result.

Correlations

		mir30a5p	MYO
mir30a5p	Pearson Correlation	1	-.109
	Sig. (2-tailed)		.482
	N	59	44
MYO	Pearson Correlation	-.109	1
	Sig. (2-tailed)	.482	
	N	44	50

Table S21. Spearman correlations between miR-155-5p and MYO in patients with HF, list of p and sig result.

Correlations

		mir155	MYO
mir155	Pearson Correlation	1	.157
	Sig. (2-tailed)		.298
	N	59	46
MYO	Pearson Correlation	.157	1
	Sig. (2-tailed)	.298	
	N	46	50

Table S22. Spearman correlations between miR-216a and MYO in patients with HF, list of p and sig result.

Correlations

		mir216a	MYO
mir216a	Pearson Correlation	1	.003
	Sig. (2-tailed)		.986
	N	59	43
MYO	Pearson Correlation	.003	1
	Sig. (2-tailed)	.986	
	N	43	50

Table S23. Spearman correlations between miR-217 and MYO in patients with HF, list of p and sig result.

Correlations

		mir217	MYO
mir217	Pearson Correlation	1	-.015
	Sig. (2-tailed)		.922
	N	59	44
MYO	Pearson Correlation	-.015	1
	Sig. (2-tailed)	.922	
	N	44	50

Table S24. Spearman correlations between miR-21-5p and HsTnT in patients with HF, list of p and sig result.

Correlations

		mir21	HsTnT
mir21	Pearson Correlation	1	.361**
	Sig. (2-tailed)		.007
	N	59	54
HsTnT	Pearson Correlation	.361**	1
	Sig. (2-tailed)	.007	
	N	54	61

**. Correlation is significant at the 0.01 level (2-tailed).

Table S25. Spearman correlations between miR-30a-3p and HsTnT in patients with HF, list of p and sig result.

Correlations

		mir30a3p	HsTnT
mir30a3p	Pearson Correlation	1	.083
	Sig. (2-tailed)		.550
	N	59	54
HsTnT	Pearson Correlation	.083	1
	Sig. (2-tailed)	.550	
	N	54	61

Table S26. Spearman correlations between miR-30a-5p and HsTnT in patients with HF, list of p and sig result.

Correlations

		mir30a5p	HsTnT
mir30a5p	Pearson Correlation	1	.229
	Sig. (2-tailed)		.092
	N	59	55
HsTnT	Pearson Correlation	.229	1
	Sig. (2-tailed)	.092	
	N	55	61

Table S27. Spearman correlations between miR-155-5p and HsTnT in patients with HF, list of p and sig result.

Correlations

		mir155	HsTnT
mir155	Pearson Correlation	1	.315*
	Sig. (2-tailed)		.019
	N	59	55
HsTnT	Pearson Correlation	.315*	1
	Sig. (2-tailed)	.019	
	N	55	61

*. Correlation is significant at the 0.05 level (2-tailed).

Table S28. Spearman correlations between miR-216a and HsTnT in patients with HF, list of p and sig result.

Correlations

		mir216a	HsTnT
mir216a	Pearson Correlation	1	.449**
	Sig. (2-tailed)		.001
	N	59	54
HsTnT	Pearson Correlation	.449**	1
	Sig. (2-tailed)	.001	
	N	54	61

**. Correlation is significant at the 0.01 level (2-tailed).

Table S29. Spearman correlations between miR-217 and HsTnT in patients with HF, list of p and sig result.

Correlations

		mir217	HsTnT
mir217	Pearson Correlation	1	.123
	Sig. (2-tailed)		.374
	N	59	54
HsTnT	Pearson Correlation	.123	1
	Sig. (2-tailed)	.374	
	N	54	61
